# Glomerular filtration rate measurement during platinum treatment for urothelial carcinoma: optimal methods for clinical practice

**DOI:** 10.1007/s10147-023-02454-3

**Published:** 2024-01-05

**Authors:** Dag Rune Stormoen, Ulla Nordström Joensen, Gedske Daugaard, Peter Oturai, Emil Hyllested, Jakob Lauritsen, Helle Pappot

**Affiliations:** 1 Department of Oncology, Rigshospitalet, Copenhagen University Hospital, Copenhagen, Denmark; 2 Department of Urology, Rigshospitalet, Copenhagen University Hospital Rigshospitalet, Copenhagen, Denmark; 3 Department of Clinical Physiology and Nuclear Medicine, Rigshospitalet, Copenhagen, Denmark; 4 Department of Clinical Medicine, University of Copenhagen, Copenhagen, Denmark

**Keywords:** Glomerular filtration rate, mGFR, eGFR, Bladder cancer, Urothelial tract cancer

## Abstract

**Background:**

We assessed the accuracy of four estimated glomerular filtration rate (eGFR) methods: MDRD, Cockcroft–Gault, CKD-EPI, and Wright.

**Method:**

The four methods were compared to measure GFR (mGFR) in patients with urothelial urinary tract cancer (T2-T4bNxMx) receiving platinum-based chemotherapy at Rigshospitalet, Copenhagen, from January 2019 to December 2021. Using standardized assays, creatinine values were measured, and mGFR was determined using Technetium-99 m diethylenetriaminepentaacetic acid (Tc-99 m-DTPA) or Cr-51-ethylenediaminetetraacetic acid (Cr-51-EDTA) plasma clearance. Patients (*n* = 146) with both mGFR and corresponding creatinine values available were included (*n* = 345 measurements).

**Results:**

The CKD-EPI method consistently demonstrated superior accuracy, with the lowest Total Deviation Index of 21.8% at baseline and 22.9% for all measurements compared to Wright (23.4% /24.1%), MDRD (26.2%/25.5%), and Cockcroft–Gault (25.x%/25.1%). Bland Altman Limits of agreement (LOA) ranged from − 32 ml/min (Cockcroft–Gault) to + 33 ml/min (MDRD), with CKD-EPI showing the narrowest LOA (− 27 ml/min to + 24 ml/min and lowest bias (0.3 ml/min). Establishing an eGFR threshold at 85 ml/min—considering both the lower limit of agreement (LOA) and the minimum cisplatin limit at 60 ml/min—allows for the safe omission of mGFR in 30% of patients in this cohort.

**Conclusion:**

CKD-EPI equation emerged as the most suitable for estimating kidney function in this patient group although not meeting benchmark criteria. We recommend its use for initial assessment and ongoing monitoring, and suggest mGFR for patients with a CKD-EPI estimated GFR below 85 ml/min. This approach could reduce costs and decrease laboratory time for 30% of our UC patients.

**Supplementary Information:**

The online version contains supplementary material available at 10.1007/s10147-023-02454-3.

## Introduction

Monitoring glomerular filtration rate (GFR) is essential when administering nephrotoxic agents like cisplatin or when drug concentration is dependent on GFR which is the case with carboplatin. GFR can be measured (mGFR) using radioactive isotopes or estimated (eGFR) from serum creatinine and clinical information. Direct measurement of GFR via glomerular filtration of radioactive or non-radioactive nucleotide tracers is precise but is time consuming and costly [1–3]. Estimated GFR from serum creatinine concentration is cheap, quick, and widely used but less accurate.

Cisplatinum-containing chemotherapy remains the backbone of treatment for patients with advanced urothelial carcinoma (UC). However, a limiting factor for this treatment is decreased kidney function. In a consensus study determining contraindications to cisplatin administration in UC, it was recommended to avoid cisplatin in patients with a GFR below 60 mL/min [4].

Carboplatin dosage is based on the Calvert equation which includes information of renal function [5]. For simplicity, eGFR is often used calculated with the Cockcroft–Gault equation [5, 6]. This method tends to overestimate GFR, and may lead to a dosing error of carboplatin by more than 20% in around one-third of patients [7]. Such dosage inaccuracies put patients at risk for reduced response rates or excess toxicity [8].

The EAU guidelines for managing metastatic and muscle-invasive bladder cancer recommend using mGFR in equivocal cases but do not specify or recommend a particular method for estimating GFR from creatinine; several are available and offer various reliability for different patient populations (Table 1) [9, 10].

**Table 1:** Overview of different measurement and estimation models to assess glomerular filtration rate. eGFR = estimated glomerular filtration rate

	Description of method	Output
Direct measurement methods		
Inulin clearance	Inulin is a polysaccharide that is freely filtered by the kidneys and neither reabsorbed nor secreted by the renal tubules. Inulin is infused into the patient’s bloodstream, and timed blood and urine samples are collected. The clearance of inulin is calculated based on the inulin concentrations in these samples. This method is considered the gold standard for GFR measurement but is labor-intensive and technically challenging	ml/min
Radiolabeled tracers	The most common radiolabeled tracers include Tc-99 m DTPA, Cr-51 EDTA, and iodine-labeled iothalamate. The clearance of these tracers is by collecting timed blood and urine samples. These methods provide accurate GFR measurements but involve the use of radioactive materials	ml/min
Estimation formulas mentioned in this paper		
Cockcroft–Gault formula: (creatinine clearance)	Age, weight, and serum creatinine level. May overestimate GFR in patients with low muscle mass	ml/min
Modification of Diet in Renal Disease (MDRD) formula (eGFR)	Age, gender, race, and serum creatinine level. The MDRD formula is more accurate than the Cockcroft–Gault formula but may underestimate GFR in patients with near-normal kidney function	mL/min/1.73m^2^
Chronic Kidney Disease Epidemiology Collaboration (CKD-EPI) formula (eGFR)	Age, gender, race, and serum creatinine level. The CKD-EPI formula provides more accurate GFR estimates across a wider range of kidney function than the MDRD formula and is currently recommended by many clinical guidelines	mL/min/1.73m^2^
Wright formula (eGFR)	Age, gender, BSA, serum creatinine	mL/min

Inulin, a natural polysaccharide freely filtered by the kidneys and not reabsorbed or secreted by the renal tubules, is the gold standard for measuring GFR [11]. However, inulin clearance is labor-intensive and is mostly replaced by the use of radiotracers. In patients diagnosed with bladder cancer, an accurate assessment of kidney function is critical owing to the nephrotoxic nature of chemotherapy treatments, coupled with the high prevalence of comorbidities such as diabetes, hypertension, sarcopenia, and obstruction of the urinary tract [12]. Given that numerous countries employ eGFR for both treatment stratification and carboplatin dosing across a variety of cancer types including urothelial carcinoma [13], it becomes imperative to compare eGFR with mGFR in this specific patient population.

This study examines a cohort of patients receiving platinum-based chemotherapy for urinary tract cancer with the aim to compare the accuracy of four eGFR formulas commonly used in clinical practice (CKD-EPI, Wright, Cockcroft–Gault, and MDRD), with measured GFR using radiotracers. Our goal was to determine the most accurate equation for eGFR and to identify situations where mGFR can be replaced by eGFR.

## Methods

This retrospective study included patients with urothelial urinary tract cancer (T2-4bNxMx) who received platinum-based treatment at the Department of Oncology, Rigshospitalet, Copenhagen, between January 2019 and December 2021. Patients were included if they had undergone at least one cycle of carboplatin or cisplatin and had an mGFR measurement with a corresponding serum creatinine value. All plasma creatinine (p-creatinine) values used in this study were measured on Roche Cobas^®^ 8000 with enzymatic determination using absorption photometry, with standardized assays, the biochemical laboratory was using international quality assurance standard DS/EN ISO 15189:2013 and has been accredited by The Danish Accreditation fund (akkr. Nr 442) [14].

Patients with a GFR above 60 ml/min received a 3-week gemcitabine–cisplatin (GC) regimen with mandatory performance status (PS) of 0–1; cisplatin on day 1, 70 mg/m^2^ and gemcitabine on day 1 and 8 (1000 mg/m^2^). Those with GFR of 50–60 ml/min received split-dose GC (cisplatin 35 mg/m^2^ at day 1 and 2). Cisplatin-ineligible patients were treated with carboplatin–gemcitabine (CaG), requiring a PS of 0–2. Carboplatin dosage was calculated based on area under the curve (AUC) 4.5 x (mGFR + 25)mg. Gemcitabine was delivered at day 1 and 8 at 1000 mg/m^2^. All palliative treatments lasted up to six cycles, while neoadjuvant GC lasted up to four cycles.

mGFR measurements were conducted at baseline, after 3rd and fifth cycle of treatment. If creatinine fell by over 25%, extra mGFR tests were conducted. Carboplatin dosage calculations relied on baseline and 4th-cycle mGFR.

For cisplatin hydration, a short regimen targeting 300 ml of diuresis was advised, requiring 3000 ml over 4 h 45 min. A few patients received a longer hydration regimen totaling 3500 ml over 6 h.

### Measurement

GFR was measured by determining the plasma clearance of Tc-99 m-DTPA. The tracer was injected intravenously in an amount of 4 MBq, and two blood samples were collected from a cubital vein 200 min later if GFR was expected to be above 30 ml/min [15]. If GFR was expected to be below 30 ml/min, four samples were drawn between 4 and 5 h after tracer injection using the 4-point method [16]. Tc-99 m plasma activity was determined using a gamma counter (Wizard 2470, PerkinElmer, Shelton, CT, USA). These GFR measurements at our institution have a variation coefficient of 10%. Some patients initiated treatment before Tc-99 m-DTPA was routinely used, or at another institution. Their GFR was measured with Cr-51-EDTA using the same technique and formulas as described for Tc-99 m-DTPA.

### GFR estimation

Baseline height and weight at each treatment occurrence were used with the corresponding creatinine value for the formulas that incorporated height and weight. BSA was determined using the Mostellers formula [17].

The time between compared serum creatinine and mGFR was limited to a maximum of 7 days at baseline and 3 days during longitudinal measurements to minimize the risk of inaccurately interpreting a high creatinine value with a mismatching mGFR. Baseline and longitudinal measurements were included. Formulas used for estimation are shown in Table S1. When comparing the formulas incorporating BSA and those standardized to 1.73 m^2^, all standardization to the surface area was removed by eGFR * BSA/1.73 m^2^. As cystatin-C is not used routinely in Denmark, we have used validated versions of the above equations, not including cystatin-C [18].

### Statistics

Patients’ demographic data were described using frequency analyses. To assess differences in GFR between hydration regimens, a Welch *t* test was applied and adjusted with the Bonferroni Holms method [19]. Normal distribution was assessed.

The accuracy of the eGFR estimation methods was evaluated by percentage of eGFR values within 10% and 30% (p10 and p30) of mGFR. These percentages were calculated for each eGFR estimation method at baseline and for all measurements.

Total Deviation Index (TDI) is a measurement of agreement between two tests and represents the deviation at which a pre-specified proportion of measurements (90%) lies. A TDI of 20% means that 90% of the measurements (eGFR) are within ± 20% of the reference value (mGFR) [20, 21]. TDI was calculated for baseline and 2nd to 4th measurement and all measurements combined.

Lin’s concordance correlation coefficients [22] (CCC) were calculated to quantify the strength, agreement, and direction of the relationship between eGFR, as estimated by the different methods, and mGFR. The statistical significance of these correlations was assessed, and the 95% confidence intervals for the correlation coefficients were calculated using 1000 bootstrap samples [22, 23]. CCC was calculated for all measurements, baseline, and 2nd to 4th measurement, and by chemotherapy agent administered. In addition, Pearson’s correlation coefficients were calculated.

A Bland–Altman analysis assessed limits of agreement between the eGFR methods and mGFR [24]. This analysis yields the mean bias (average difference between eGFR and mGFR), the standard deviation of the bias, and the limits of agreement (LOAs) for each method. The LOAs provide an interval within which 95% of the differences between the two measurements fall and were computed as the mean bias ± 1.96 standard deviations. A mixed effects model for repeated measurements, was applied for the Bland–Altman analysis, to examine for random effects, and if this model did not differ from the traditional Bland–Altman, we decided to use the traditional calculation for easier interpretability.

Scatterplots with linear regression (mGFR on x-axis, eGFR on y-axis) was performed using R^2^ to evaluate the fit of the model and presented for visual interpretation of the data, a linear regression line is fitted to represent the correlation, and a 45-degree angle is applied to illustrate closeness of plots to Lin’s concordance correlation coefficient (CCC).

All statistical analyses and graphs were performed using R v.4.2.0 [25], and a *p* value of < 0.05 was considered statistically significant.

## Results

We identified 151 individuals undergoing treatment with CG or CaG for muscle invasive (neoadjuvant) or metastatic (palliative) urothelial cell cancer. Five patients were excluded from GFR analyses due to a gap of over 7 days between creatinine and mGFR measurements at baseline, additionally 29 measurements were excluded for missing p-creatinine value within the pre-specified time between mGFR and p-creatinine. Consequently, the analysis was based on 146 patients with 345 mGFR measurements with corresponding p-creatinine values. (Table 2) Median body surface area (BSA) was 1.97 m^2^ and median body mass index (BMI) was 25.7 kg/m^2^. We did not observe a weight loss during treatment (Figure S5).

**Table 2:** Patient characteristics

Characteristic	Total *N* (%)
Total number of patients	146 (100.0%)
Gender	
Female	48 (32.9%)
Male	98 (67.1%)
ECOG PS	
0	97 (66.4%)
1	46 (31.5%)
2	3 (2.1%)
Body weight kg—median (range)	78.5 (47.2–131.9)
Body height cm—median (range)	175 (149–191)
Body surface area (m^2^)—median (range)	1.97 (1.46–2.59)
Body mass index (kg/m^2^)—median (range)	25.7 (16.7–43.1)
Smoker	
Current smoker	46 (31.5%)
Former smoker	71 (48.6%)
Never smoker	27 (18.5%)
Unknown	2 (1.4%)
Kidney function at baseline (by mGFR)	
> 90 ml/min	53 (36.3%)
60-89 ml/min	56 (38.4%)
30-59 ml/min	36 (24.7%)
15-29 ml/min	1 (0.7%)
Measurement method	Number of mGFR measurements
Cr-51-EDTA	124 (33.2%)
Tc-99-DTPA	250 (66.8%)
Total	374
Chemotherapy treatment (baseline)	n patients (planned)
Adj carbo/gemc 4 cycles	2 (1.4%)
Downstaging cis/gemc 6 cycles	9 (6.2%)
Neoadjuvant cis/gemc 4 cycles	51 (34.9%)
Palliative carbo/gemc 6 cycles	38 (26.0%)
Palliative cis/gemc 6 cycles	42 (28.8%)
Split course Palliative cis/gemc 6 cycles	4 (2.7%)
Total	146 (100%)

Of the 345 treatment cycles included in analysis, 118 cycles were CaG without a particular hydration regimen, for CG: 208 cycles consisted of 4-h short hydration regimen, 11 cycles with 6-h long regimen, and 8 cycles using the split-course regimen. There were no statistical differences between mGFR (or eGFR) between the hydration groups both at baseline and for all measurements, although small sample sizes for long/split should be noted.

The mean values over the treatment period, for mGFR and eGFR, are depicted in Figure S1.

At the baseline, the p10 values were highest for CKD-EPI (47%) and lowest for Cockcroft–Gault (37%) (Table 3). However p30 was comparable across all eGFR: Cockcroft–Gault (88%), Wright (87%), CKD-EPI (90%), and MDRD (88%). (Table 3) A similar pattern was seen for all measurements. (Table 4).

**Table 3:** Representation of statistic divided by measurement number, baseline is 1st measurement, 2nd to 4th measurement; 5th to 8th measurement is excluded as only 12 patients have this many measurements

	Measurement	MDRD	Cockcroft–Gault	CKD-EPI	Wright	No. of patients
LOA ml/min	Baseline	− 28.5, 33.3	− 32.3, 26.1	− 27.6, 23.8	− 25.1, 29.9	146
	2nd to 4th	− 24.3 33.6	− 29.8 30.8	− 26.3, 30.5	− 21.6, 33.3	108
CCC (95% CI)	Baseline	0.84 (0.78–0.88)	0.85 (0.80–0.89)	0.87 (0.82–0.90)	0.86 (0.81–0.89)	146
	2nd to 4th	0.76 (0.70–0.82)	0.77 (0.70–0.82)	0.78 (0.71–0.83)	0.76 (0.70–0.82)	108
TDI (95% CI)	Baseline	26.24% (21.68–30.91)	25.06% (22.44–27.56)	21.80% (19.20–24.38)	23.41% (20.31–26.45)	146
	2nd to 4th	25.46% (22.18–28.72)	25.46% (22.07–28.88)	24.08% (20.80–27.34)	24.97% (21.89–28.14)	108
p10/p30	Baseline	41.78%/88.36%	36.99%/87.67%	47.26%/89.73%	42.47%/86.99%	146
	2nd to 4th	36.5%/83.7%	33.7%/83.7%	37.1%/85.3%	39.3%/81.4%	108

Parenthesis contains 95% confidence interval; for limits of agreement, parenthesis contain bias. p10/p30 denotes percentage of 10% and 30% deviance from mGFR, respectively. Table include both cisplatin- and carboplatin-treated patients

*LOA* 95% limits of agreement from Bland–Altman calculation. *CCC* Lin’s concordance correlation coefficient. *TDI* 90% Total Deviation Index

**Table 4:** Statistics for all measurements combined

	MDRD	Cockcroft–Gault	CKD-EPI	Wright	Optimal model
LOA (bias) ml/min	− 25.94, 33.10 (3.58)	− 30.9, 28.6 (− 1.15)	− 27.03, 27.64 (0.30)	− 23.1, 31.8 (4.35)	− 6, 6 (0)
CCC (95% CI)	0.815 (0.78–0.85)	0.82 (0.78–0.85)	0.83 (0.79–0.86)	0.82 (0.78–0.85)	> 0.9
TDI (95% CI)	25.5% (22.9–28.0)	25.1% (23.1–27.2)	22.9% (21.0–25.0)	24.1% (21.9–26.3)	< 10%
p10/p30	38.26%/85.80%	34.49%/85.22%	40.00%/87.25%	40.00%/ 83.77%	> 90%/100%

Parenthesis contains 95% confidence interval; for limits of agreement, parenthesis contain bias. Optimal model (that does not exist) by recommendations from [21, 22]. Table includes both cisplatin- and carboplatin-treated patients

*LOA* Bland–Altman’s limit of agreement, *TDI* Total Deviation Index, *CCC* Lin’s concordance correlation coefficient, *LOA-MEM* limits of agreement calculated using mixed effects model for repeated measurement

From the Bland–Altman analyses and the 95% limits of agreement (LOA), the bias observed for MDRD, Cockcroft–Gault, CKD-EPI, and Wright were 3.6 ml/min, − 1.2 ml/min, 0.3 ml/min, and 4.4 ml/min, respectively. The LOA span for these equations ranged from lower LOA at − 30.9 ml/min for Cockcroft–Gault to upper LOA at 31.8 ml/min for Wright (Table 4, baseline values shown in Table 3, Figures S1 and S2). When adjustments were made using a mixed effects model accounting for repeated measurements, for the 95% LOA, biases changed only slightly (data not shown).

By setting a threshold of 85 ml/min for eGFR (lower LOA for CKD-EPI plus lower recommended lowest GFR for cisplatin at 60 ml/min [4]), 97 patients had an eGFR (CKD-EPI) above 85 ml/min, constituting 28.1% (95% CI 23.4–33.2%) of the measurements. By appending this cutoff to our dataset, using CKD-EPI, four measurements (out of 345) are slightly below the cisplatin threshold of 60 (range of mGFR 55–58), making it clinically feasible to deliver full-dose cisplatin still (Table S2).

The Total Deviation Index (TDI) at baseline for MDRD, Cockcroft–Gault, CKD-EPI, and Wright were 26%, 25%, 23%, and 24%, respectively (Tables 3 and 4 for later measurements).

**Fig. 1:**
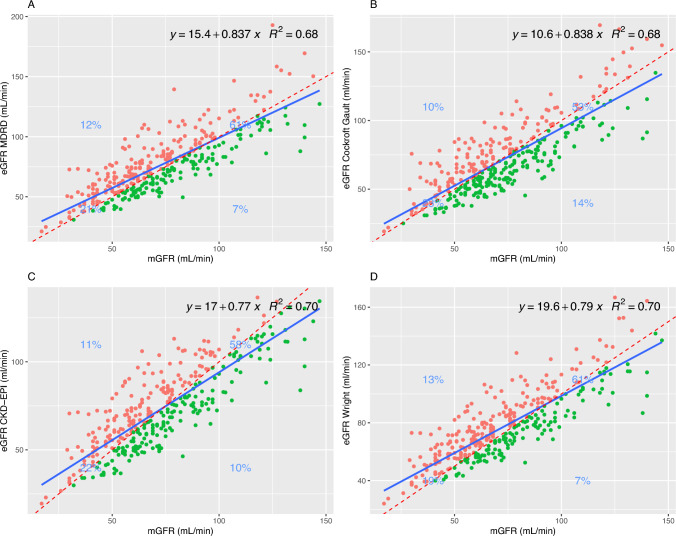
Scatter plot of mGFR vs eGFR by **A** MDRD, **B** Cockcroft–Gault, **C** CKD-EPI, **D** Wright. Regression line with intercept, coefficient and R-squared in top right corner of each scatter plot. Green line represents cutoff at 60 mL/min and percentages in each graph quadrant represent total % of measurements within quadrant representing agreement (upper right and lower left) between methods. All 345 measurements are plotted. Dotted-red line represents perfect alignment, and blue line represents best linear regression line

The CCC suggested good agreement for all methods with MDRD, Cockcroft–Gault, and Wright all showing a CCC of approximately 0.82, and CKD-EPI slightly higher at 0.83 for all measurements, and in line with TDI, CCC improves at baseline for CKD-EPI to 0.87 (0.82–0.90), (all with a *p* < 0.001). Pearson’s correlation coefficients similarly shows that all four equations had strong linear relationships with the reference values (depicted in Figs. 1, S1S4).

Lastly, for p10 and p30, MDRD presented values of 38.26% and 85.80%, Cockcroft–Gault 34.49% and 85.22%, CKD-EPI reported 40.00% and 87.25%, and Wright 40.00% and 83.77%, respectively. At baseline, p10/p30, similarly to TDI and CCC, improves slightly (Table 3). Notably, when divided by chemotherapy, estimations in later cycles retained their accuracy in the carboplatin-treated group, but diminished in the cisplatin group. (Tables S4 and S5).

## Discussion

We compared the GFR measured using Tc-99 m-DTPA or Cr-51-EDTA radiotracers as gold standard with the estimated GFR derived from four distinct formulas. Our results indicate that the CKD-EPI equation performed best among these four. For patients diagnosed with urothelial tract carcinoma, the meticulous assessment of renal function is imperative. This necessity arises from the elevated risk of treatment-induced nephrotoxicity as well as the intrinsic likelihood of tumor-mediated obstruction within the urinary system. Either condition poses a significant risk of irreversible renal failure.

While all four eGFR equations presented strong CCC with mGFR, there were variations in their biases, wide limits of agreement, and TDIs. All indicates substantial variability in the estimation of GFR, also evident by the TDI above 20%. Despite the variability and bias in the eGFR estimation methods when compared to mGFR, the CKD-EPI method displayed the strongest results compared with mGFR at baseline and for all measurements, the highest percentage of eGFR values within 10%, and 30% accuracy of mGFR, the highest CCC, the lowest TDI, and the slightest average error (bias) in eGFR estimation for baseline and all measurements combined.

The characteristics of the patients in our study (Table 2) align well with the patient profiles presented in the existing bladder cancer literature [26]. The patients underwent multiple GFR measurements throughout their treatment period, which enabled us to evaluate the consistency during treatment.

Our results mimic previous GFR studies, where the selected eGFR equations demonstrated a strong positive correlation with mGFR, although conclusions are disparate depending on the patient population [2, 8, 18, 27–34]. In a study examining testicular cancer patients receiving cisplatin, a high correlation was observed between eGFR (creatinine clearance) and mGFR both at baseline and 3-month post-treatment. However, during the treatment period, this correlation was not evident, a phenomenon that may be attributable to cisplatin nephrotoxicity, and also the significant weight loss observed during treatment, with an average loss of approximately 10 kg. [35] In our cohort, the weight remained stable during treatment and was comparable to the baseline weight (Figure S5). The less stringent and less effective administration of anti-emetic therapy during platinum-based treatments in 1988, as opposed to contemporary practices, may account for the observed absence of weight loss within our cohort. Nevertheless, there was a diminution in the accuracy of the estimation methods during the treatment phase for cisplatin-treated patients. This decrease of correlation was not as marked as in [35], and the methods retained reliability for clinical use during treatment. (Table 3, S4 and S5). Notably, this deterioration was not evident in the carboplatin-treated group, likely due to carboplatin being less nephrotoxic.

The measurement inaccuracy of EDTA/DPTA mGFR is shown to be within 5–15% compared to inulin, with a tendency to underestimate GFR [36–38], and a variation between mGFR and eGFR of 15% could be argued to be acceptable accuracy for eGFR equations [21].

CCC indicated a good but suboptimal concordance between measurements, with CKD-EPI showing the best but declining performance in later measurements for the cisplatin-treated part of the cohort. [21, 22] Later measurements revealed lower mGFR values, suggesting eGFR’s decreased accuracy in kidney failure due to various factors such as chemotherapy side effects. TDI values observed in this study exceeded those of the established gold standard, [21] although current KDIGO guidelines recognize the presence of within-person variation in measured GFR (mGFR) [38]. The literature has yet to reach a consensus on an acceptable level of agreement between mGFR and eGFR, as evidenced by the ongoing debate in recent publications [21, 39, 40].

Surface-normalized GFR standardizes for body size (1.73 m^2^), whereas formulas like Cockcroft–Gault or Wright inherently adjust for it [34, 41]. Given that chemotherapy dosage relies on non-normalized GFR, we propose that laboratory reports should adjust the eGFR with the patient’s BSA for those formulas not accounting for BSA, presenting both surface normalized and non-normalized eGFR, echoing previous suggestions regarding abandonment of BSA indexing [41–43]. This study employed enzymatically measured creatinine from accredited laboratories. Enhanced accuracy in such modern creatinine assays has improved eGFR equation reliability, even when these equations were initially based on older assays with greater variability [44]. Even with optimal assays, there is an inherent variability of creatinine based on factors such as muscle mass, protein intake, sarcopenia, and chronic illnesses (diabetes mellitus, hypertension, etc.) that complicates creatinine-based eGFR [38, 45]. In addition, the reliability of the eGFR equations varies among different ethnic groups. Studies have indicated that the equation tends to underestimate eGFR in some ethnic groups, such as African Americans, if specific ethnic coefficients are not applied [46]. Conversely, it may overestimate eGFR in other populations, including some Asian cohorts where modified equations are in use [47]. These discrepancies arise from variations in muscle mass, dietary protein intake, and other genetic and environmental factors that influence serum creatinine levels.

In the lack of a more stable and freely filtered serum marker than creatinine, knowledge of the inaccuracies of eGFR, and when to choose mGFR, has important clinical implications. The nephrotoxic effects of agents such as cisplatin may exacerbate the discrepancy between plasma creatinine levels and the GFR, as GFR may decline while plasma creatinine remains unchanged. This trend was evident in our cohort, with a decrease in the CCC (Table S3) in patients treated with cisplatin. In contrast, such a trend was not observed in patients who were administered carboplatin (Table S4).

The study’s finding suggesting CKD-EPI is the most precise method aligns with similar studies for urothelial carcinoma using different mGFR references (CrCl), although agreement is not consistent for all cancers [8, 18, 38, 48, 49]. We found no clinical characteristics, baseline creatinine values or treatment related characteristics that helps to guide the use of mGFR or eGFR for individual patients, except for the decline in accuracy during later cycles of cisplatin treatment, which we attribute to the nephrotoxic effect of cisplatin. Even for patients with a low GFR, the variability in LOA is as consistent as for the higher GFRs (Figures S2 and S3). This suggests that fluctuations in creatinine that do not reflect actual GFR changes affects the variation in agreement between eGFR and mGFR, partly this variation can be driven by cisplatin nephrotoxicity in the longitudinal measurements. On the other hand, the within-subject variation of mGFR of 15% reflects actual GFR changes and also affects the inconsistency in agreement [38]. Care should be taken when interpreting eGFR on patients with extreme values of BMI, and in the last cycles of cisplatin treatment [18, 38, 41].

As in other comparative studies, this study affirms the good performance of the CKD-EPI equation on enzymatically measured creatinine [8, 10, 38, 48, 49]. Clinical decisions on cisplatin eligibility and carboplatin dose calculation should be guided by standard protocols, with mGFR remaining accessible, particularly for patients receiving cisplatin. Presently, guidelines lack clarity and do not favor one method over another. We suggest an update in clinical guidelines for managing urothelial carcinoma, to recommend CKD-EPI as the preferred equation for eGFR echoing the recommendation of KDIGO, although we do acknowledge that the differences between the estimation methods are modest [50]. Exclusive reliance on eGFR to determine glomerular filtration rate may lead to an elevated risk of adverse events, especially in patients receiving cisplatin during subsequent cycles of treatment.

Strengths and limitations

A strength of this study is the large dataset with corresponding mGFR from a clinical gold standard reference and serum creatinine values from an accredited laboratory using enzymatic assays and well annotated patient cohort. The estimation methods do not meet optimal standards, but a larger cohort would not amend this and further comparative studies using the same methods for this patient population are unlikely to yield different results. Limitations of this study include its retrospective design with possible confounders unaccounted for such as actual changes in GFR between eGFR and mGFR if not measured in the same day. Inherent variabilities in reference measurements (mGFR) and external factors like day-to-day variations in performance of laboratory equipment and variations in patient conditions can influence results. Lacking an absolute eGFR gold standard achieving specific LOA, CCC, and TDI benchmarks, we focus on relative differences between available methods.

## Conclusion

The CKD-EPI equation outperformed other tested eGFR equations but did not meet criteria of optimal agreement with mGFR. Prudence is warranted in the application of any eGFR equation during the advanced cycles of cisplatin treatment. We suggest using Tc-99 m-DTPA for patients with CKD-EPI eGFR below 85 ml/min. For patients with eGFR > 85 ml/min, CKD-EPI can monitor kidney function, applying mGFR only if a significant decrease in eGFR occurs (i.e., > 25%). This approach could constitute a rational recommendation in guidelines and reducing the need for mGFR would cut costs and lessen workload while easing the burdens for some patients during treatment.

### Supplementary Information

Below is the link to the electronic supplementary material.

Supplementary file1 (DOCX 1103 KB)

## Data Availability

Data will be shared upon reasonable request to corresponding author.
