# Supplementary material for: Glomerular filtration rate measurement during platinum treatment for urothelial carcinoma: optimal methods for clinical practice
Source: Int J Clin Oncol. 2024 Jan 5;29(3):309–17. doi: 10.1007/s10147-023-02454-3 (PMC10884137; doi:10.1007/s10147-023-02454-3)
Supplement: Supplementary file 1 — Supplementary file1 (DOCX 1103 KB) [file 10147_2023_2454_MOESM1_ESM.docx]

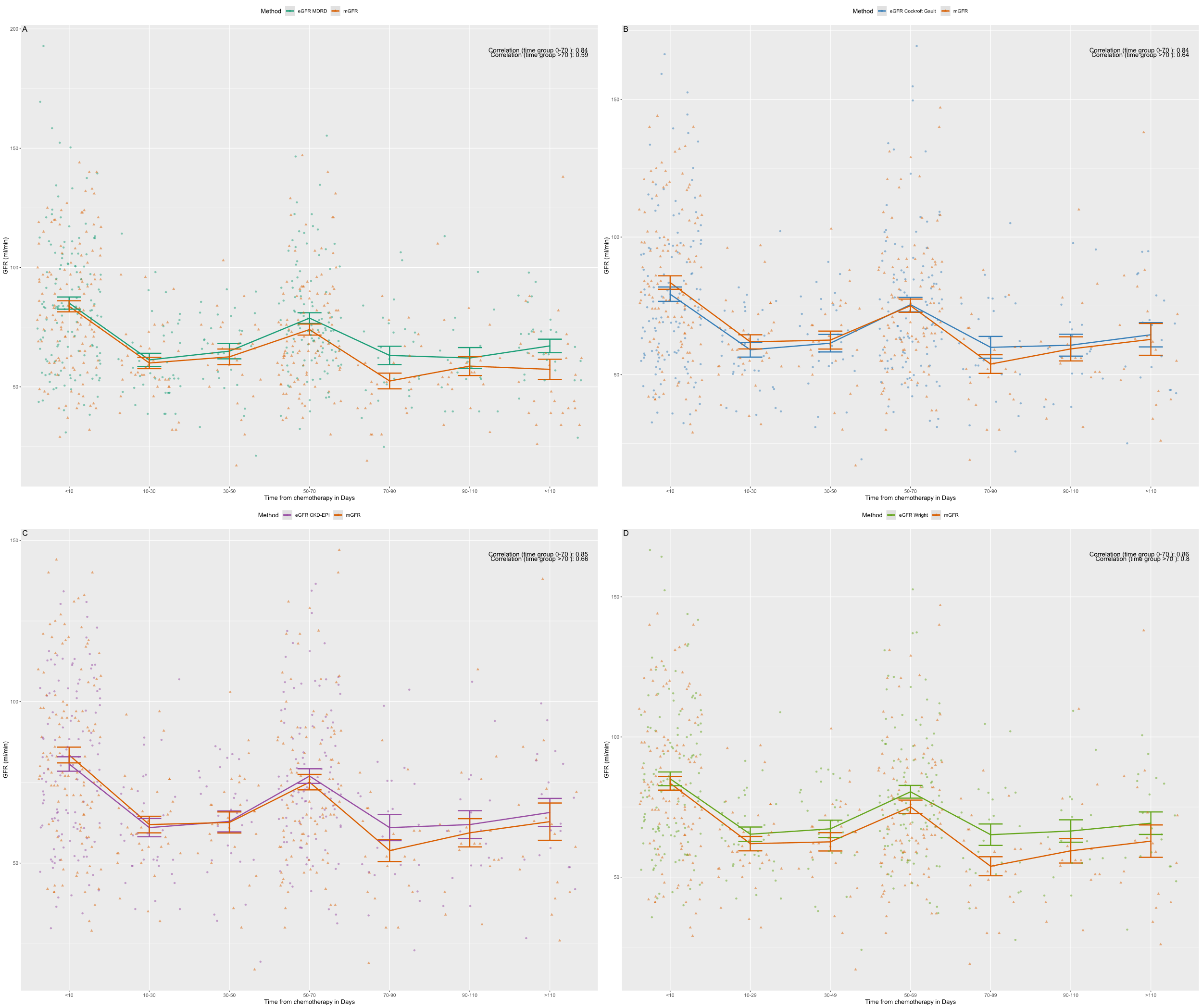


Figure S1: mGFR and eGFR by different formulas plotted longitudinally with Pearson's correlation for agreement between eGFR and mGFR within <70 days from chemo and >70 days from chemo (top-right corner of each graph). A: MDRD calculated eGFR, B: Cockroft-Gault calculated eGFR, C: CKD-EPI calculated eGFR, D: Wright calculated eGFR. All surface normalization has been removed for formulas standardized to a surface area of 1.73m^2^. All correlations are highly statistically significant (top right corner of each graph).


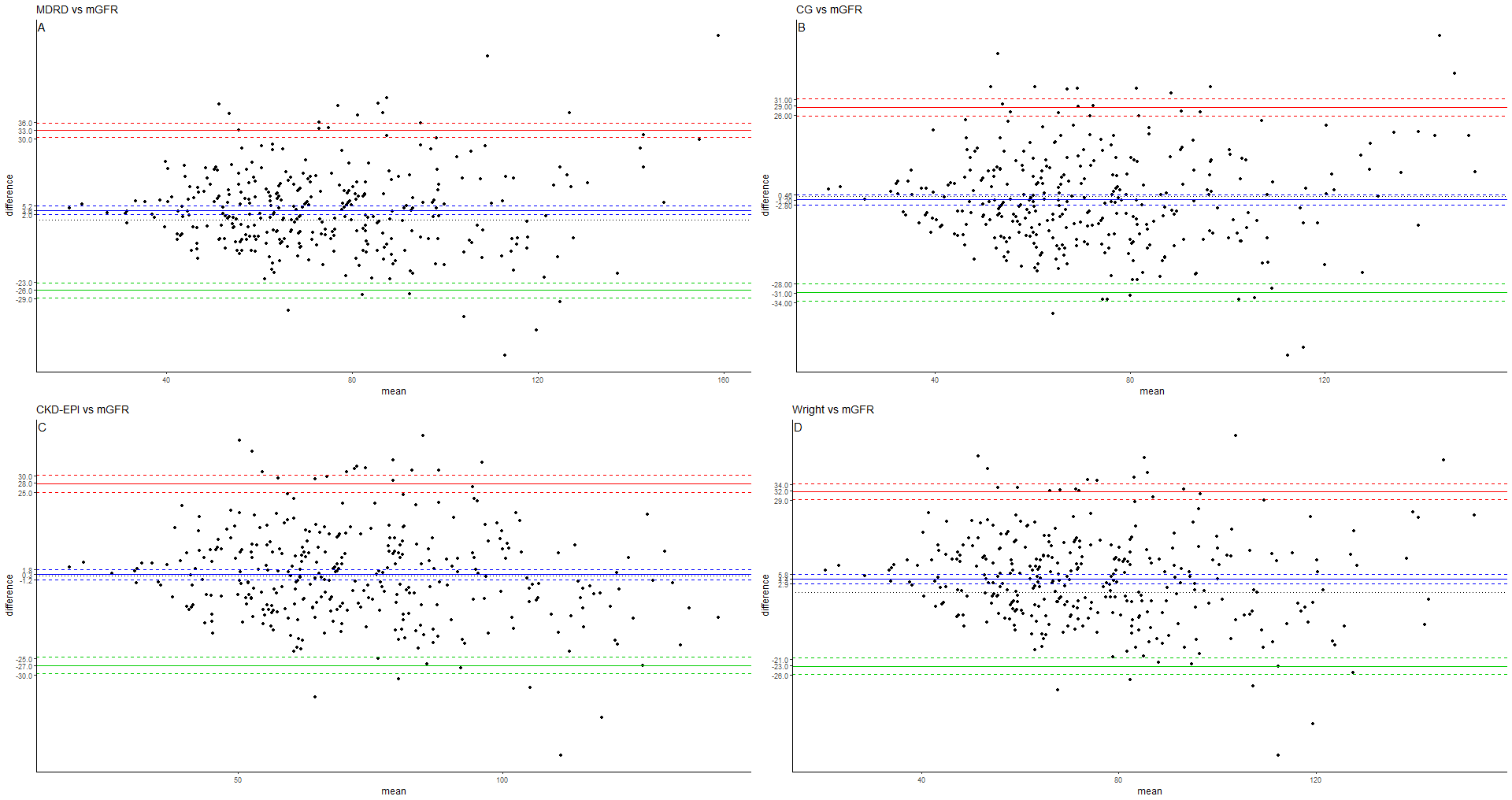


Figure S2: All measurements: Bland-Altmann plots showing mean of measurements eGFR and mGFR by difference between measurements. A: eGFR calculated by MDRD and mGFR, B: eGFR calculated by Cockroft Gault and mGFR, C: eGFR calculated by CKD-EPI and mGFR, D: eGFR calculated by Wright and mGFR. Dashed lines represent 95% Confidence intervals.


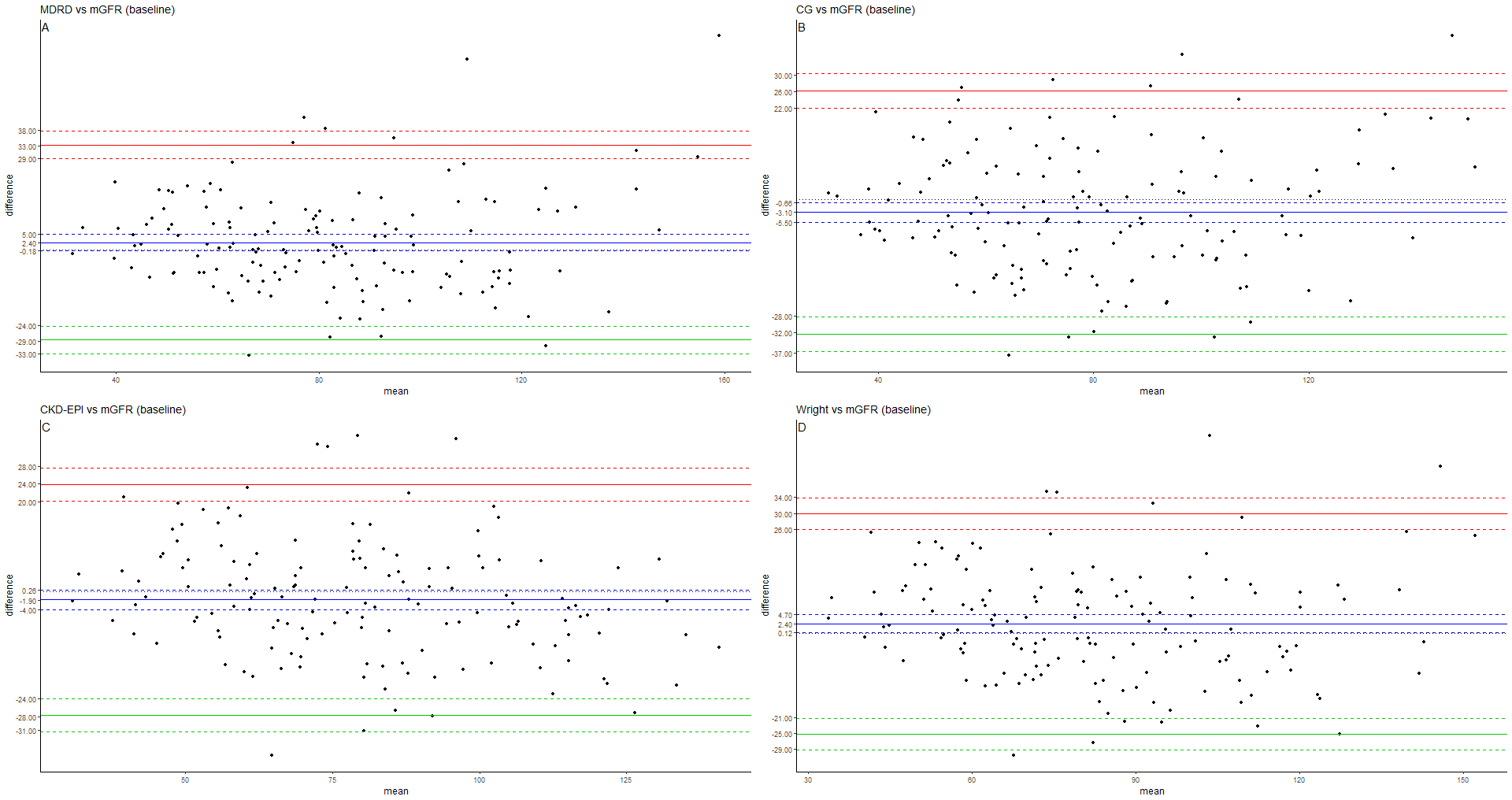


Figure S3 : Bland altman plot of baseline values of eGFR vs mGFR for the four different equations. A: eGFR calculated by MDRD and mGFR, B: eGFR calculated by Cockroft Gault and mGFR, C: eGFR calculated by CKD-EPI and mGFR, D: eGFR calculated by Wright and mGFR. Dashed lines represent 95% Confidence intervals.


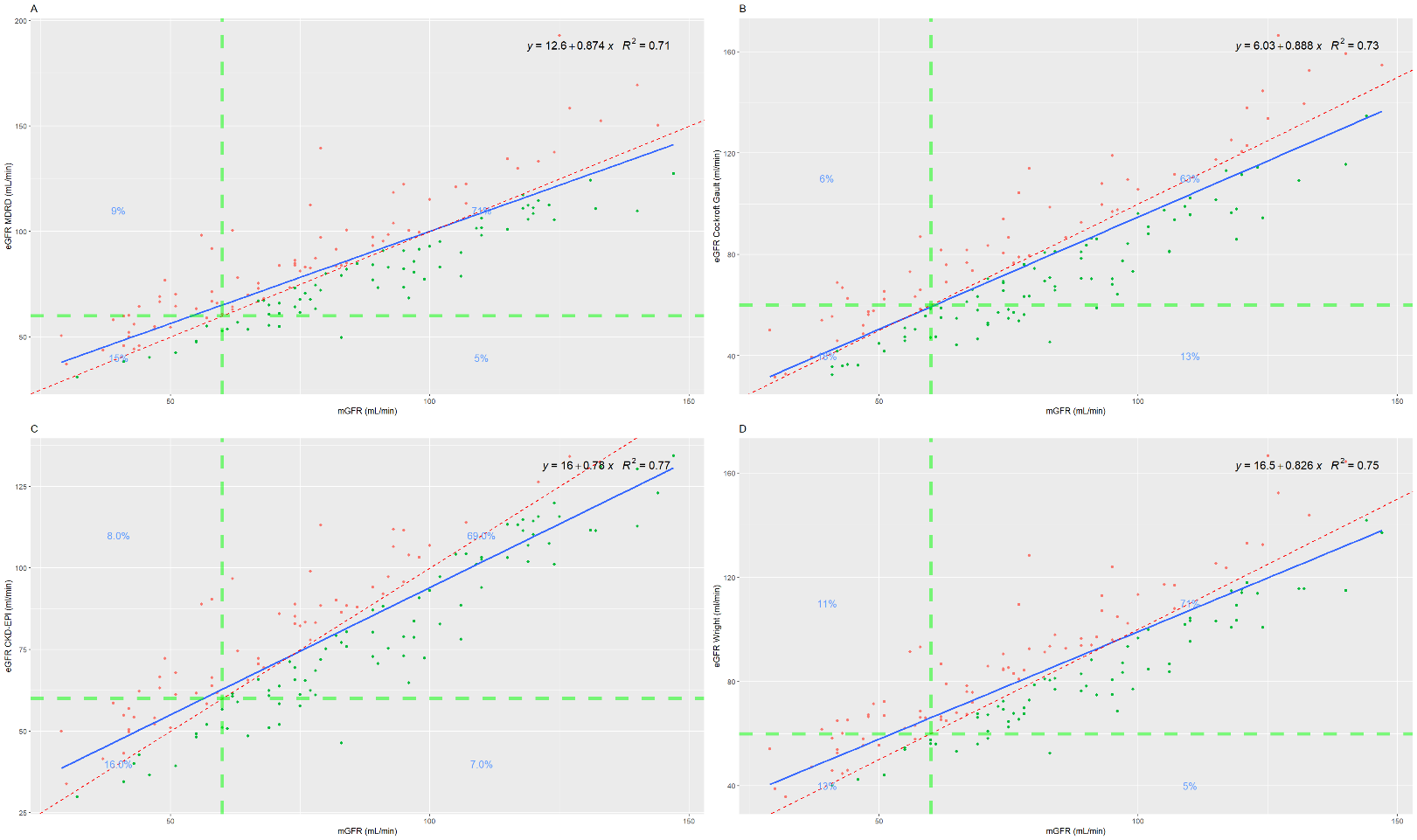


Figure S4: Baseline values of eGFR vs mGFR by A: MDRD, B: Cockroft Gault, C: CKD-EPI, D: Wright. Regression line with intercept, coefficient and R-squared in top right corner of each XY-plot. Green line represents cut-off at 60mL/min and percentages in each graph quadrant represents total % of measurements within quadrant representing agreement (upper right and lower left) between methods. Dotted-red line represents perfect alignment, blue line represent best linear regression line.

**
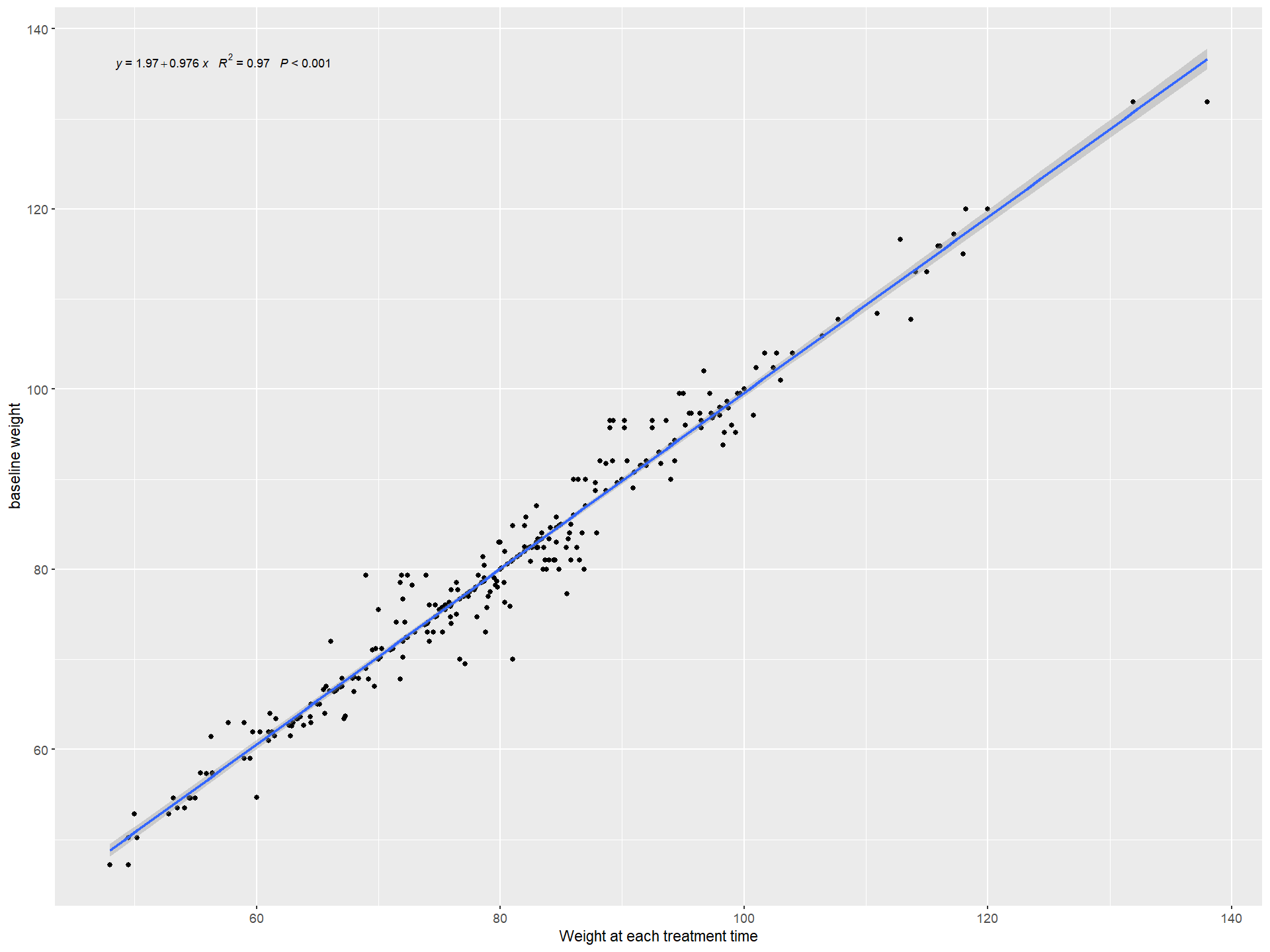
**

**Figure S5,** Baseline weight for each patient (Y-axis) against longitudinal weights for each patient showing a good linear relationship with little discrepancy between longitudinal weight and baseline weight with R-squared at 0.97

| Table S1 |  |  |
| --- | --- | --- |
| MDRD | eGFR(mL/min/1.73 m2)=175×(SCr×0.0113)−1.154 × Age−0.203 ×(0.742  if  female) ×(1.212  if  African American) |  |
| Cockroft-Gault | eCrCl(mL/min)=(140−Age)×Weight×(1−0.15×Sex)/72×SCr×0.0113 | Sex = 1 if female, 0 if male. |
| Wright enzymatic | eGFR(mL/min)=(6230−32.8×age)×BSA×(1−0.23×Sex)/SCr |  |
| CKD-EPI | eGFR(mL/min/1.73 m2)= 141×min((SCr×0.0113)/κ, 1)α × max((SCr×0.0113)/κ,1)−1.209 ×0.993Age×1.018[ if  female]×1.159[ if  black] | κ is 0.7 for females and 0.9 for males, α is –0.329 for females and –0.411 for males, min indicates the minimum of SCr × 0.0113/κ or 1, and max indicates the maximum of SCr × 0.0113/κ or 1 |

Table S1 : Formulas used for estimating GFR.

| **Table S2** |  |  |  |  |  |  |  |  |
| --- | --- | --- | --- | --- | --- | --- | --- | --- |
| **age** | **crea** | **sex** | ***mGFR*** | ***BSA*** | **eGFR MDRD (mL/min)** | **eGFR Cockroft Gault (ml/min)** | **eGFR CKD-EPI (ml/min)** | **eGFR Wright (ml/min)** |
| 67 | 81 | M | 56 | 1.77 | 89.68 | 73.5 | 88.20 | 88.12 |
| 74 | 74 | M | 56 | 1.78 | 98.10 | 73.1 | 88.88 | 91.47 |
| 74 | 92 | M | 55 | 2.12 | 90.88 | 83.7 | 86.22 | 87.63 |
| 65 | 94 | M | 58 | 2.14 | 91.87 | 86.9 | 90.33 | 93.29 |

Table S2, 4 patients with estimated GFR of >85, and mGFR < 60 but still close to 60, making cisplatin's delivery feasible to these patients.

| **Chemo cycle number** | **Mean Cisplatin Dose** | **Mean mGFR (ml/min)** | **Mean eGFR CKD-EPI (ml/min)** | **Mean eGFR Cockroft-Gault (ml/min)** | **CCC**  **mGFR vs CKD-EPI** | **CCC mGFR vs Cockroft-gault** |
| --- | --- | --- | --- | --- | --- | --- |
| 1 | 130.0 | 88.1 | 85.8 | 82.6 | 0.84 | 0.78 |
| 2 | 98.0 | 58.7 | 58.5 | 57.3 | 0.67 | 0.68 |
| 3 | 109.4 | 68.8 | 72.4 | 71.9 | 0.74 | 0.71 |
| 4 | 107.2 | 74.6 | 78.7 | 75.4 | 0.61 | 0.52 |

Table S3, Cisplatin treated patients, with an overview of the first four cycles, mean cisplatin dose, mean mGFR, mean estimated GFR for CKD-EPI and Cockroft-Gault and Concordance Correlation Coefficient (CCC) at each treatment cycle.

| **Chemo cycle number** | **Mean Carboplatin Dose** | **Mean mGFR (ml/min)** | **Mean eGFR CKD-EPI (ml/min)** | **Mean eGFR Cockroft-Gault (ml/min)** | **CCC**  **mGFR vs CKD-EPI** | **CCC mGFR vs Cockroft-gault** |
| --- | --- | --- | --- | --- | --- | --- |
| 1 | 325.4 | 64.6 | 66.4 | 64.6 | 0.84 | 0.84 |
| 2 | 286.4 | 60.4 | 56.3 | 54.6 | 0.86 | 0.83 |
| 3 | 281.4 | 57.0 | 59.5 | 56.6 | 0.81 | 0.81 |
| 4 | 270.6 | 67.8 | 70.4 | 70.8 | 0.90 | 0.92 |

Table S4: Carboplatin treated patients, with overview of the first four cycles, mean carboplatin dose, mean mGFR, mean estimated GFR for CKD-EPI and Cockroft-Gault and Concordance Correlation Coefficient (CCC) at each treatment cycle.
